#  Enhancement Effect of Trypsin on Permeation of Clindamycin Phosphate Through Third-degree Burn Eschar 

**Published:** 2013

**Authors:** Azadeh Ghaffari, Ali Manafi, Hamid Reza Moghimi

**Affiliations:** a *Department of Pharmaceutics, School of Pharmacy, Shahid Beheshti University of Medical Sciences, Tehran, Iran*; b *Department of Surgery, School of Medicine, Tehran University of Medical Sciences, Tehran, Iran. *

**Keywords:** Burn eschar, Skin, Clindamycin phosphate, Permeation enhancers, Trypsin.

## Abstract

Antimicrobial therapy remains to be the most important method of wound infection treatment. Systemically administered antimicrobials may not achieve therapeutic level in wound. On the other hand, in the absence of surgical debridement (due to any reason), most topically applied antimicrobials cannot penetrate the wound in therapeutic amount due to the presence of eschar. Burn eschar is a proteinous structure with some lipid components in which proteins seems to play an important role in the barrier effects of eschar. Therefore, in this study the effect of protein-acting enhancer (trypsin) on permeation of hydrophilic model drug (clindamycin phosphate) was investigated.

To perform this investigation, permeation of saturated clindamycin phosphate was studied at 32°C through trypsin-treated and untreated eschar samples for 12 h using home-made static diffusion cells. Third-degree burn eschar samples were separated at the time of surgical debridement (7-14 days post burn) from burned patients. Before each experiment, eschar was hydrated for 12 h and samples were then treated with trypsin solution (1%, w/v) for 4 and 24 h. Clindamycin was measured by a HPLC method developed here.

Results showed that after trypsin-treatment for 4 and 24 h, clindamycin phosphate permeation flux was increased significantly by about 1.5 and 2 times and permeation lag-time was decreased by about 2 and 1.3 times respectively.

The present results show that permeation of drugs through burn eschar can be increased considerably by trypsin.

## Introduction

Thermal destruction of the skin barrier and formation of avascular necrotic tissue (eschar), that is suitable environment for microbial colonization and proliferation, besides depression of local and systemic host cellular and humoral immune responses, can potentially cause morbidity and mortality in burn patient through sepsis (1). 

To prevent sepsis, one of the important strategies is to control burn wound infection, of which, topical usage of antibiotics is very crucial. To prevent bacterial or fungal invasion, the ability of antibiotics to penetrate the burn eschar and beneath the eschar, where the microorganisms may proliferate and invade next tissue, is very important (1, 2). Previous investigations have shown that most of the antibiotics used in burn patients topically could not penetrate the burn eschar in therapeutic amount (1-3). Eschar debridement has been used to remove eschar barrier and solve the mentioned problem, but debridement surgery is not possible for a lot of the third-degree burned patients (4). So, for better drug permeation through third-degree burn eschar, in our laboratories, application of different enhancers on barrier performance of third-degree burn eschar has been investigated and it has been shown that permeation of drugs through burn eschar can be improved by chemical enhancement methods like terpenes, glycine and water (3-6).

Burn eschar is a proteinous structure with some lipid component (7). Our previous investigations have revealed that both lipid and protein domains might play important roles in barrier performance of eschar (6). The present investigation aims to overcome the barrier effects of eschar through altering the protein domain. It has been shown that trypsin can improve eschar debridement through protein effect (8). Therefore, it was decided here to investigate the effect of trypsin on permeation of a hydrophilic model drug through third degree burn eschar. Hydrophilic drugs are expected to prefer the protein domain for permeation. Clindamycin phosphate with molecular weight of 504.9 and log *P *(octanol/water) of 0.5 (9) was chosen as a model hydrophilic drug for this investigation.

To the best of our knowledge, there is nodata available in the literature about effect of trypsin or other protein acting enzymes on the permeation of drugs through burn eschar.

## Experimental

*Chemicals*

Clindamycin phosphate was purchased from Suzhou Pharmaceutical Factory (China). Monobasic potassium phosphate (98-100.5%), sodium hydroxide (> 98%), phosphoric acid (85-88%), HPLC-grade acetonitrile and trypsin (pancreas protease, 200 FIP-U/g EC 3.4.21.4) were purchased from Merck (Germany).

*Eschar samples*

Third-degree burn eschar samples, which were separated at the time of surgical debridement (1–2 weeks post-burn) from burned patients, were obtained from Motahari Burn Center (Tehran, Iran). The cause of burning in all patients was flame. Eschar samples were from 4 patients, 3 men and 1woman (35 ± 22 years, mean ± SD). Thickness of eschar samples was measured to be 1.5 ± 0.6 mm (mean ± SD). Large pieces of eschars were stored at -20⁰C until use; not later than 12 months. Our studies have shown that barrier properties of eschar do not change during the mentioned storage conditions.

*Permeation studies*

Permeation of clindamycin phosphate was studied through trypsin-treated and untreated eschar samples. For permeation studies, large pieces of burn eschar were thawed at ambient temperature and cut into appropriate smaller pieces. The eschar samples were then fully hydrated by placing the samples in water for 12 h at ambient temperature. Permeation studies were performed using home-made diffusion cells with effective surface area of approximately 1.8 cm^2^. Eschar samples were placed between donor and receptor chambers of the cells while the epidermal side faced the donor compartment. 

The cells were then placed in a thermostatically controlled water bath with stirrer. The temperature was kept at (37°C ± 0.5) in the receptor chamber that gives a temperature of approximately 32°C at the surface of the eschar. The speed of stirring in the receptor chamber was 300 rpm.

It has been shown that aqueous solution of trypsin (1% w/v) for 24 h has improved wound debridement (8), therefore, a 24 h treatment time was chosen here. Besides, to investigate the possibility of application of lower treatment times, a lower exposure time of 4 h was also investigated here. For trypsin treatment, the receptor phase of the diffusion cell was filled with 25 milliliters of phosphate buffer solution pH = 7.4 (10) and the donor phase of the diffusion cell was filled by 3 milliliters of trypsin 1% (w/v) in the same buffer solution. For control samples, donor and receptor phases of diffusion cells were filled with the same phosphate buffer solution. The cells (control and test) were then placed in a thermostatically controlled water bath for 4 and 24 h. To reduce biological variability, control and test samples were chosen from same piece of eschar.

After trypsin treatment, both phases of the diffusion cells were emptied and washed with water. Receptor and donor phases were then filled with 25 mL phosphate buffer solution pH = 7.4 and saturated clindamycin phosphate in phosphate buffer solution pH = 7.4 respectively and this time point was considered as time zero. Cells were put in a thermostatically controlled water bath with stirrer as described previously.

Serial samples were collected from the receptor chamber for 12 h and their clindamycin phosphate contents were analyzed by HPLC as described later. Sink condition was maintained at all the times of the experiments.

The cumulative amount of permeated clindamycin phosphate was plotted against time and the slope and intercept of the linear portion of the plot was measured as the steady state flux (J, mg cm^-2^h) and lag-time (L, h) respectively. Permeability coefficient (Kp) was then calculated using J and donor drug concentration (C) by Fick’s law (Kp = J/C).

The effects of trypsin on permeation flux and lag-time were measured as ratio of trypsin-treated over control (untreated) data.

Data were found to be normal by Kolmogrov-Smirnov and Shapiro-Wilk tests. Therefore, Permeation flux and lag-time were statistically compared using t-test. The level of significance was set at p < 0.05. The statistical analysis was computed with the SPSS software version 17.0 (SPSS Inc., Chicago).

*Solubility studies*

Saturated solutions of clindamycin phosphate in phosphate buffer solution pH = 7.4 at 32ºC were prepared by adding excess amount of drug to the phosphate buffer solution and stirring for 24 h at room temperature and then, 24 h at 32 °C. After this period, the excess drugs were filtered using PTFE 0.45 μm membrane filter (Chromafil^®^Xtra PTFE-45/25 Macherey-Nagel GmbH & Co. KG, Germany) and solutions were diluted and analyzed by HPLC as described below.

*HPLC analysis of clindamycin phosphate*

Clindamycin phosphate was measured by a modified HPLC method suggested by USP (10). Samples were analyzed by HPLC apparatus (Merck), using a 25 cm ×4.6 mm RP-18 column with 5 micrometer particle size (Perfectsil Target ODS-3, MZ-Analysentechnik). The mobile phase was acetonitrile and pH 2.5 phosphate buffer (22.5:77.5, v/v). The flow-rate was 1 mL/min, and clindamycin phosphate was detected by UV detector at a wavelength of 210 nm. Results showed a linear relationship (r^2^ = 0.995) between area under the curve and the concentration of clindamycin phosphate in the range of 0.01–10 mg/mL. Recovery percentage and inter-day and intra-day studies revealed good accuracy and repeatability of this method.

*Sample preparation for HPLC analysis*

As the eschar samples release traces of proteins in the receptor phase, it was necessary to precipitate these proteins before injection to HPLC. Acidic protein precipitation was done for samples by adding 20 μL of perchloric acid 60% (v/v) to 0.5 mL of sample followed by vortexing for 5 min and centrifugation at 9000×g for 10 min. The supernatant was then used for drug analysis.

*Method validation for sample preparation*

To evaluate possible interaction of the material released from the eschar with the assay methods, control permeation experiment (using donor phases without drugs) were performed as explained above and the corresponding receptor phases were analyzed by HPLC by clindamycin phosphate assay method. The obtained chromatograms did not show any peak at the place where clindamycin phosphate appears.

Also samples taken from the receptor phases of the control experiments (using donor phases without drugs) were spiked with clindamycin phosphate standard solution at 0.1 mg/mL. These solutions were then treated for protein precipitation as mentioned before and recoveries of the drugs were measured afterwards. The recovery of clindamycin phosphate was calculated to be 93-98% (n = 9).

## Result and Discussion

*Solubility studies*

The solubility of clindamycin phosphate in phosphate buffer solution at 32°C was measured to be 84.9 ± 9.8 mg/mL (n = 6), that is very close to what is reported by Resman *et al*., 80 mg/mL (11), although higher solubilities are also reported in the literature (12).

*Permeation studies*

Clindamycin phosphate permeation data through control and 4 h trypsin-treated tissues are shown in Table 1. Permeation flux and lag-time of clindamycin phosphate from saturated aqueous solution through untreated burn eschar were calculated to be 1.40 mg cm^-2^h and 1.27 hr respectively. Trypsin treatment for 4 h increased permeation flux significantly by about 1.5 times (p < 0.005) and decreased permeation lag-time by about 2 times, that was not statistically significant (Table 1).

**Table 1:** Permeation flux and lag-time of clindamycin phosphate through burn eschar treated by trypsin for 4 h. Data are mean ± SD, number = 4-5.

Treatment	Permeability				Lag-time		
	Flux (mg cm^-2^h)	K_P_× 10^3^ (mg h^-1^)	Ratio^a^	p-value^b^	Lag-time (h)	Ratio^a^	p-value^b^
Control	1.40 ± 0.22	16.27 ± 2.65	1	-	1.27 ± 0.63	1	-
Trypsin	2.04 ± 0.23	23.75 ± 2.70	1.46	0.004	0.57 ± 0.54	0.45	0.122

^a^Trypsin/Contro

^b^Two-sided t-test

Trypsin treatment for 24 h also increased permeation flux of clindamycin phosphate through burn eschar significantly; by about 2 times (p < 0.05). The lag-time was decreased by about 1.3 times at the same condition, but this decrease was not statistically significant..

 It worth to note that after trypsin treatment of burn eschar for 24 h, 2 (out of 7) of eschars samples were seen to be perforated. Eschar perforation was not observed in lower trypsin treatment duration of 4 h. Permeation data of these samples are not provided here.

 The above mentioned results clearly show that trypsin is able to increase permeation of clindamycin phosphate, and possibly other drugs, through burn eschar. The lag-time, that is an indication of therapeutic effect onset time, was also showed a decrement trend, but the effect was not statistically significant, that should be due to complexity of eschar, as, it has been shown that non-barrier components in complex membranes affect lag-time more than flux (13, 14).

 As is shown in Tables 1 and 2, clindamycin phosphate permeation flux and lag-time through control samples of two sets of studies are different. Differences between permeation flux and lag-time of different eschars are due to biological variations of burn eschars. Therefore, to reduce the effect of such unavoidable variations on the results, control and test study of each set of experiment were done on one burn eschar sample here. 

 Burn eschar is a proteinous structure with some lipid component (7). As trypsin affects proteins, it may be concluded that proteins play an important role in the barrier effect of eschar toward hydrophilic drugs. 

**Table 2:** Permeation flux and lag-time of clindamycin phosphate through burn eschar treated by trypsin for 24 h. Data are mean ± SD, number = 5.

Treatment	Permeability				Lag-time		
	Flux (mg cm^-2^h)	K_P_× 10^3^ (mg h^-1^)	Ratio^a^	p-value^b^	Lag-time (h)	Ratio^a^	p-value^b^
Control	0.60 ± 0.13	7.03 ± 1.47	1	-	2.00 ± 0.82	1	-
Trypsin	1.35 ± 0.68	15.92 ± 8.02	2.25	0.041	1.52 ± 1.09	0.76	0.531

^a^Trypsin/Control

^b^Two-sided t-test

**Figure 1:**
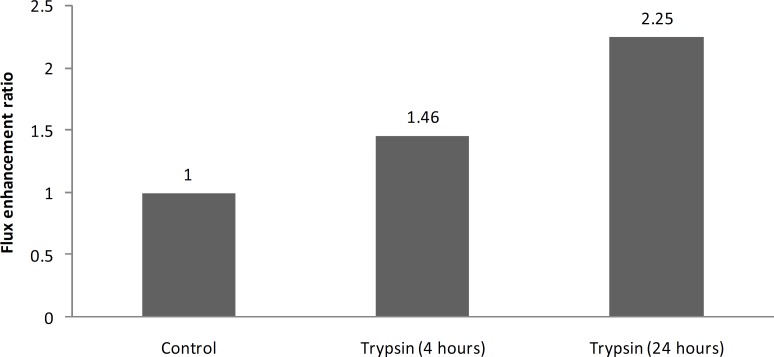
Enhancement effect of trypsin toward permeation of clindamycin phosphate through third-degree burn eschar treated for 4 and 24 h

The present results also show that by increasing the trypsin treatment time from 4 h to 24 h, enhancement effect of trypsin increases from 1.4 to 2.2 and it shows that enhancement effect of trypsin on burn eschar is time dependant (Figure 1). 

Trypsin is a large proteinous enzyme (molecular weight, 23.3 KDa). It has been shown that large molecules are not able to penetrate normal intact burn eschar (15). Therefore the present results might show that trypsin enhances its own permeation and by increasing the time of trypsin treatment, more digestion could happen and therefore, hence more enhancement effect of trypsin. 

Our previous studies showed that although, permeation of lipophilic drugs (nitroglycerine and silver sulfadiazine) were increased by enhancers like glycerine, water, sodium lauryl sulphate, that are expected to alter proteins, permeation of hydrophilic drug (chlorhexidine) was not increased by these enhancers (6). The present results show that trypsin, which digests burn eschar proteinous structure and is used for eschar debridement (8), is able to increase permeation of hydrophilic drugs through burn eschar. 

## Conclusion

The present results show that proteins play an important role in the barrier effect of eschar to hydrophilic drugs and permeation of these drugs through burn eschar can be increased considerably by trypsin. This strategy might be able to improve wound antimicrobial therapy. Based on the current data, a minimum treatment time of 4 h might be required; at least until effectiveness of lower treatment times are investigated. Beside this, the method might require other adjustments based on the cause of burn or patient’s condition. 
